# Structurally simple synthetic 1, 4-disubstituted piperidines with high selectivity for resistant *Plasmodium falciparum*

**DOI:** 10.1186/s40360-018-0233-2

**Published:** 2018-07-04

**Authors:** Moses N. Ngemenya, Grace Ntube Abwenzoh, Hermia Nalova Ikome, Denis Zofou, Fidele Ntie-Kang, Simon M. N. Efange

**Affiliations:** 1 Biotechnology Unit and Department of Biochemistry and Molecular Biology, Faculty of Science, University of Buea, Buea, Cameroon; 2 Department of Biochemistry and Molecular Biology, Faculty of Science, University of Buea, Buea, Cameroon; 3 Department of Chemistry, Faculty of Science, University of Buea, Buea, Cameroon

**Keywords:** Resistance, Antiplasmodial, Piperidines, Cytotoxicity, Selectivity

## Abstract

**Background:**

Emergence of resistance to artemisinins and some of their combinations in chemotherapy of clinical malaria has intensified the search for novel safe efficacious antimalarial molecules. Fourteen synthetic 1, 4-disubstituted piperidines with simple molecular structures were evaluated in this study.

**Methods:**

Antiplasmodial activity were determined against cultured chloroquine-sensitive 3D7 and resistant Dd2 strains of *P. falciparum* by in vitro parasite growth inhibition. A primary screen was done to identify active compounds by fluorescence microscopy followed by a secondary screen to determine IC_50_ and IC_90_ values of active compounds by the parasite lactate dehydrogenase assay. Cytotoxicity of active compounds was assessed using the MTT/formazan assay and selectivity indices (SIs) determined. Optical densities were analysed to obtain experimental results.

**Results:**

The compounds produced 56 to 93% inhibition of parasite growth at 40 μg/mL. Eight compounds (2 ketone, 5 alcohol and one amine analogues) showed high activity (IC_50_s between 1 and 5 μg/mL). Nine compounds were highly selective for the parasite (SIs = 15 to 182). Three promising (alcohol) analogues were identified: [1-(4-fluorobenzyl) piperidin-4-yl] [4-fluorophenyl] methanol, (**7**), [1-(3, 4-dichlorobenzyl) piperidin-4-yl] [4- fluorophenyl] methanol **(8)** and [1-(4-bromobenzyl) piperidin-4-yl] [4- fluorophenyl] methanol **(11)** which were more active on the resistant strain (IC_50_ values between 1.03 to 2.52 μg/mL), than the sensitive strain (IC_50_ values between 2.51 to 4.43 μg/mL).

**Conclusions:**

The alcohol analogues were the most active and most selective for the parasite with three promising hit molecules identified among them, suggesting the hydroxyl group at C-7’ in these alcohol analogues is contributing greatly to their antiplasmodial activity. Further exploration of the core structure using chemistry approaches and biological screening including in vivo studies in an animal model of malaria may yield important antimalarial leads.

## Background

Parasite resistance in chemotherapy of clinical malaria is a major threat to treatment outcome. The antimalarials frequently used include the 4–aminoquinolines, arylamino alcohols, antifolates and the artemisinins in various formulations. Artemisinin-based combination therapies (ACTs) along with other control tools have greatly reduced malaria morbidity and mortality in the last decade [1]. There is increasing resistance to the other drug classes cited above while delayed parasite clearance to the artemisinins has been detected in five countries of the Greater Mekong, in Southeast Asia along with multi-drug resistance in *P. falciparum*. In this area delayed parasite clearance has also been reported for ACTs where there is resistance to the partner drug and it is thought that artemisinin could facilitate selection for resistance to the partner drug [2, 3].

The approaches presently used to discover new antimalarials include screening of synthetic molecules generated using medicinal chemistry and natural products from plants, chemical modification of existing antimalarials, development of hybrid molecules with a combination of suitable pharmacochemical properties; testing of commercially available drugs approved for treatment of other human diseases and molecular modelling using virtual screening technology and docking [4, 5]. Methods presently employed in compound screening include high-throughput screens of large compound libraries in phenotypic (whole- cell) or target assays. Whole cell screens are done on blood, liver and transmission stages of the parasite [4].

The piperidines constitute a very large chemical class of both natural and synthetic compounds containing the six membered heterocyclic nucleus [6]. The nucleus is present in many naturally occurring alkaloids [7]. This nucleus confers significant biological properties hence the compounds are of tremendous importance in medicinal chemistry. Extensive synthetic exploration of piperidine–containing molecules has yielded very diverse structural analogues with wide ranging and interesting pharmacological activities [8]. Piperdine is one of the most common heterocycles found in pharmaceutical agents, typically as a linker or to improve the drug's pharmacokinetic profile [9]. Hence the pharmacological spectrum of the analogues include anti-allergic, anti-inflammatory, analgesic, antioxidant, anti-psychotic, antidepressant, anti-diabetic, anticancer, antibacterial, antimalarial, antifungal and other activities with a considerable number of compounds in clinical use [10, 11].

In a previous communication, we reported the synthesis of a series of 1, 4-disubstituted piperidines. When tested, these compounds displayed high affinity for sigma receptors. In a bid to further explore the pharmacological potential of these compounds and in view of the wide range of biological activities reported for substituted piperidines, these 1, 4-piperidines were also screened for antiplasmodial activity as part of our continuing search for efficacious antimalarials [12].

## Methods

### Synthetic piperidines

**Fig. 1:**
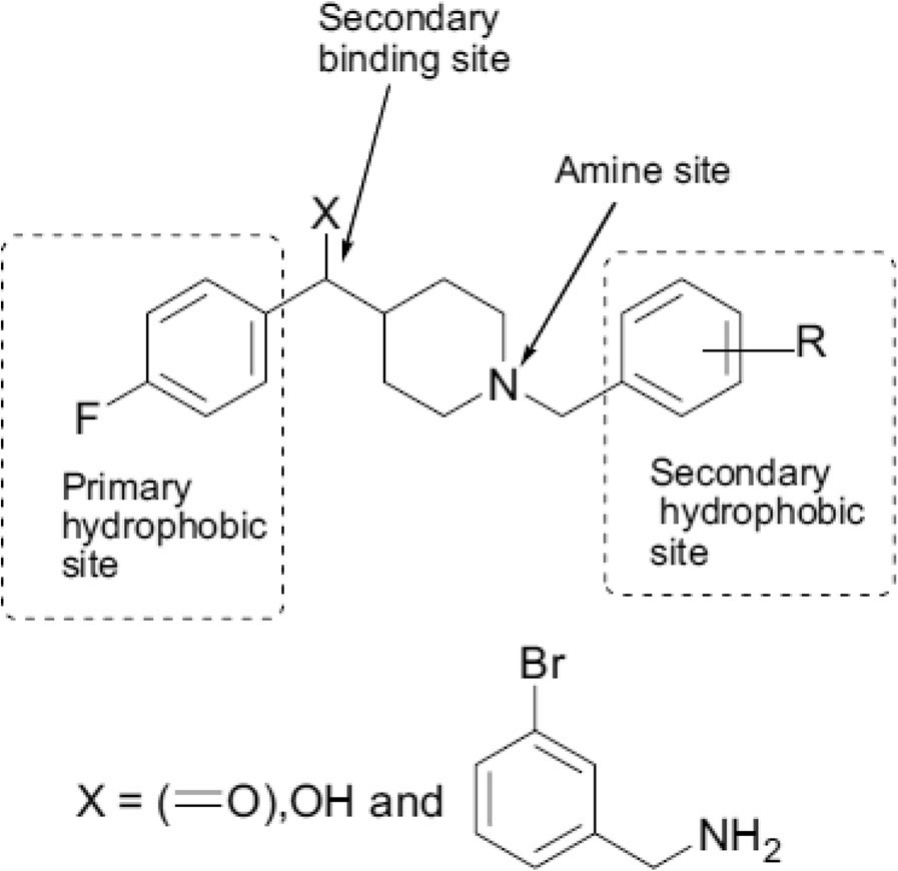
Parent structure of 1, 4-disubstituted piperidine analogues showing important features

The synthesis of the 14 piperidines has been described in detail [12]. The compounds are based on a common parent skeleton shown in Fig. 1 with substituents at the C4 and N1 positions of the piperidyl parent fragment. The compounds can be grouped into three chemical classes based on the functional groups present in these substituents: ketones (**1**–**6**), alcohols (**7**–**12**) and amines (**13**, **14**). For the bioassay all compounds were tested in the hydrochloride form. The ketones: [1-(4-fluorobenzyl) piperidin-4-yl] [4-fluorophenyl] methanone (**1**), [1-(3, 4-dichlorobenzyl) piperidin-4-yl] [4-fluorophenyl] methanone (**2**), [1-(4-chlorobenzyl) piperidin-4-yl] [4-fluorophenyl] methanone (**3**), [1-(2-nitrobenzyl) piperidin-4-yl] [4-fluorophenyl] methanone (**4**), [1-(4-bromobenzyl) piperidin-4-yl] [4-fluorophenyl] methanone (**5**), [1-(4-methylbenzyl) piperidin-4-yl] [4-fluorophenyl] methanone (**6**); methanol analogues: [1-(4-fluorobenzyl) piperidin-4-yl] [4-fluorophenyl] methanol (**7**), [1-(3, 4-dichlorobenzyl) piperidin-4-yl] [4- fluorophenyl] methanol (**8**), [1-(4-chlorobenzyl) piperidin-4-yl] [4-fluorophenyl] methanol **(33c)** (**9**), [1-(2-nitrobenzyl) piperidin-4-yl] [4- fluorophenyl] methanol (**10**), [1-(4-bromobenzyl) piperidin-4-yl] [4- fluorophenyl] methanol (**11**), [1-(4-methylbenzyl) piperidin-4-yl] [4- fluorophenyl] methanol (**12**); and two bromobenzylamine analogues: N-{[1-(2-nitrorobenzyl) piperidin-4-yl] [4-fluorophenyl] methyl}-3-bromobenzylamine (**13**) and N-{[1-(4-bromobenzyl) piperidin-4-yl] [4-fluorophenyl] methyl}-3-bromobenzylamine (**14**).

### In vitro growth inhibition assay

Two *Plasmodium falciparum* parasite strains were obtained from MR4 (Manassas, Virginia, USA) and stored in liquid nitrogen. The 3D7 (MRA-102) strain is chloroquine- sensitive while Dd2 is a multidrug resistant clone of the W2 strain with resistance to chloroquine, quinine, mefloquine, sulfadoxine and pyrimethamine. Parasite stocks were thawed, grown and maintained in continuous culture by the candle jar technique with some modifications [13, 14]. The culture consisted of 4% hematocrit suspension of human O+ erythrocytes in RPMI-1640 medium (10.43 g/L supplemented with 0.01 mg/mL gentamycin, 25 mM Hepes buffer, 25 mM NaHCO_3_) and 5 g Albumax II. All culture reagents were obtained from Sigma-Aldrich Inc. (Germany) except for Albumax II (Gibco; Invitrogen, USA). Cultures were incubated at 37 °C in 5% CO_2_.

A primary screen for antiplasmodial activity at a single concentration was performed against Dd2 as described with some modifications [15], to identify active compounds. Stock solutions of compound (1 mg/mL) was initially dissolved in dimethylsulfoxide (DMSO), then gentamycin-free incomplete medium (without Albumax II) added giving 2% final DMSO; the solutions were sterilized and stored. The positive control solutions (artemether and quinine at 5 μg/mL) were prepared similarly. The compounds were tested at a final concentration of 40 μg/mL in duplicate in a 96 well microtitre plate (100 μL per well), followed by 100 μL of 1% parasitized blood. Positive and negative controls (no drug) were included. The plate was incubated under the same culture conditions as above for 48 h and antiplasmodial activity determined by fluorescence microscopy (Leitz Wetzlar, Germany) with acridine orange stain at × 40 magnification under oil immersion [14]. The number of infected erythrocytes (*Ei*) was counted in 20 monolayer fields (approximately 1500 cells per field) and the percentage parasitaemia was calculated using the formula:

1 $$ \%\kern0.5em \mathrm{parasitaemia}=\frac{\sum {E}_i}{30,000}\times 100 $$

The percentage inhibition per concentration was then obtained using the formula below:

2 $$ \mathrm{percentage}\ \left(\%\right)\kern0.5em \mathrm{inhibition}=\frac{\%\mathrm{parasitaemia}\ \mathrm{of}\ \mathrm{control}\ \mathrm{well}\hbox{-} \%\mathrm{parasitaemia}\ \mathrm{of}\ \mathrm{test}\ \mathrm{well}}{\%\mathrm{parasitaemia}\ \mathrm{of}\ \mathrm{control}\ \mathrm{well}}\times 100 $$

A secondary screen was performed as above for compounds that showed greater than 50% inhibition in the primary screen at final concentrations from 0.625–40 μg/mL in order to determine the IC_50_ and IC_90_ values on 3D7 and Dd2 strains; IC_50_ values of controls were also determined similarly. After incubation the plate was frozen at − 20 °C for the parasite lactate dehydrogenase (pLDH) assay.

### Parasite growth quantification by lactate dehydrogenase assay

The reagent preparation and assay were done as described [16]. The plates were subjected to 3 freeze-thaw cycles to haemolyze the red blood cells. One hundred microlitre (100 μL) of Malstat reagent was added to each required well of a fresh microtitre plate followed by 20 μL of culture. Then 25 μL of nitroblue tetrazolium/phenazine ethosulfate (NBT/PES) solution was added initiating the reaction. The plates were then incubated for an hour in the dark and optical density (OD) measured at 650 nm (Emax-Molecular Devices Corporation, California, USA).

### Cytotoxicity test

This was done on monkey kidney epithelial cells, LLC-MK2, (ATCC, USA) as described [17] except for use of same culture medium and conditions for *P falciparum* above. The cells, 10,000 in 100 μL medium per well, were cultured for 24 h to become confluent and attached to the bottom of the well and the medium replaced with 100 μL of fresh medium. Solutions of 9 compounds (1000 μg/mL) which showed moderate to high antiplasmodial activity, prepared same as in the primary screen above, were serially diluted with medium and added in duplicate wells to final concentrations of 7.8 to 500 μg/mL. A negative control without test substance was included. The plates were incubated for 72 h at 37 ^o^ C in 5% CO_2_ and cell viability were determined using microscopy to observe dead cells which appeared dark and deformed. This was followed by the MTT/formazan assay as described above with slight modification [17]. The CC_50_ values (50% cytotoxic concentration) for each compound was obtained using Microsoft excel 2007. The selectivity index (*SI*) was calculated using the formula:

3 $$ SI=\frac{{\mathrm{CC}}_{50}\ \mathrm{on}\ \mathrm{LLC}\hbox{-} \mathrm{MK}2\ \mathrm{cells}\ }{{\mathrm{IC}}_{50}\ \mathrm{on}\ \mathrm{P}.\mathrm{falciparum}} $$

### Data analysis

For the parasite lactate dehydrogenase assay, the background OD of blank wells containing non parasitized red blood cells was subtracted from values of all test wells. OD values from control wells void of test compound represented the maximum pLDH activity. The OD values obtained were used to determine the IC_50,_ and IC_90_ values using the software IC Method IC Estimator V1.2 [18], an online calculator of IC values. Each assay was done twice in duplicates and the 4 IC_50_ or CC_50_ values obtained were pooled together and expressed as mean IC ± S.D.

## Results

### Antiplasmodial activity of piperidines

Screening for antiplasmodial activity involved a two-stage process, primary and secondary. Only compounds that displayed greater than 50% inhibition of *P. falciparum* growth in the primary screen progressed to the secondary assay. In the current study, 13 out of 14 compounds met this criterion while one compound, **4**, displayed poor inhibition of *P. falciparum* growth (Table 1) and was eliminated from further screening. In the secondary assay, the following criteria were adopted for antiplasmodial activity: active, IC_50_ < 5 μg/mL; moderately active: 5 μg/mL ≤ IC_50_ ≤ 10 μg/mL; weakly active: IC_50_ ≥ 10 μg/mL. All 13 compounds displayed dose dependent activity on both the 3D7 and Dd2 strains. For most of the compounds, the IC_50_ values were comparable between the chloroquine-sensitive 3D7 and -resistant Dd2 strains, spanning a range of IC_50_ values between 1.03 and 14.51 μg/mL (Table 2). Consequently, the rank orders of potency were fairly similar between the two strains. On the 3D7 strain, eight compounds (**2**, **3**, **7**, **8**, **9**, **11**, **12**, **14**) emerged as active, while two (**1**, **13**) were moderately active and three (**5**, **6**, **10**) were classified as weakly active. On the Dd2, seven compounds (**3**, **7**, **8**, **9**, **11**, **12**, **14**) emerged as active while there were three each in the moderately active (**1**, **2**, **13**) and weakly active (**5**, **6**, **10**) groups. Compounds **9** and **11** displayed the highest activities against both *P. falciparum* strains, with IC_50_ values of 1.53 μg/mL and 1.03 μg/ml, respectively, but these compounds were about one order of magnitude less potent than quinine. Three compounds (**7**, **8**, **11**) were particularly noteworthy because they were more potent on the chloroquine-resistant Dd2 strain than the drug-sensitive 3D7 strain, suggesting they could serve as useful leads for the discovery of new antimalarials.

**Table 1:** Inhibition of *P. falciparum* Dd2 growth by piperidine analogues at 40 μg/mL

Chemical class	Code	Substituent	Inhibition (%)
Ketones	1	4”-F	83.2
	2	3”, 4”-Cl_2_	82.7
	3	4”-Cl	56.6
	4	2”-NO_2_	27.7
	5	4”-Br	64.8
	6	4”-Me	59.1
Alcohols	7	4”-F	81.3
	8	3”, 4”-Cl_2_	81.7
	9	4”-Cl	93.2
	10	2”-NO_2_	79.7
	11	4”-Br	85.6
	12	4”-Me	83.1
Amines	13	2”-NO_2_	72.9
	14	4”-Br	86.0

Dd2 is a chloroquine-resistant *P. falciparum* strain

**Table 2:** Antiplasmodial activity of 1, 4-disubstituted piperidines on *P. falciparum* 3D7 and Dd2 strains

Compound code	IC values (μg/mL)			
	3D7		Dd2	
	IC_50_	IC_90_	IC_50_	IC_90_
1	8.45 ± 2.11	17.85 ± 0.18	5.69 ± 2.38	27.59 ± 0.78
2	4.30 ± 3.75	17.61 ± 0.06	5.97 ± 0.02	24.86 ± 4.14
3	3.21 ± 0.21	17.9 ± 2.39	4.12 ± 1.28	32.38 ± 0.67
4	ND	ND	ND	ND
5	10.02 ± 0.30	17.23 ± 0.97	14.51 ± 1.34	32.31 ± 4.17
6	13.63 ± 0.31	19.08 ± 2.46	12.75 ± 3.21	37.15 ± 0.53
7	4.00 ± 0.67	14.61 ± 1.52	^a^2.52 ± 0.77	17.71 ± 4.34
8	4.43 ± 0.32	13.89 ± 1.25	^a^1.30 ± 0.47	17.33 ± 3.27
9	1.53 ± 0.24	16.08 ± 2.13	1.67 ± 0.24	16.08 ± 2.13
10	13.54 ± 0.54	19.11 ± 0.02	13.98 ± 1.76	35.28 ± 2.02
11	2.51 ± 0.30	16.80 ± 0.88	^a^1.03 ± 0.124	27.82 ± 0.98
12	3.37 ± 0.82	15.74 ± 0.00	2.78 ± 0.09	10.2 ± 3.31
13	5.88 ± 2.49	8.94 ± 2.50	5.38 ± 0.69	25.93 ± 2.83
14	2.28 ± 0.64	11.31 ± 3.50	2.57 ± 1.64	16.21 ± 0.35
Art	0.045	–	0.039 ± 0.00	0.046 ± 0.00
QN	0.171	–	0.127	–

3D7 is a chloroquine-sensitive *P. falciparum* strain. -Not done, Art artemether, QN quinine. ND not determined due to low activity in the primary screen. Chemical classes of compounds:- carbonyls:**1**–**6**; alcohols: **7**–**12**, and amines: **13**–**14**. ^a^Compounds more active on chloroquine-resistant Dd2 than 3D7

### Cytotoxicity of piperidines

The 10 compounds which showed moderate to high activity also recorded high CC_50_ values ranging from 62 to 375 μg/mL i.e. from 2 to 12 times above the cut-off point for cytotoxicity of 30 μg/mL [19] suggesting the more active compounds are non-cytotoxic. For 3D7, compounds **3** and **14** had the lowest and highest *SI* values of 19 and 163 respectively while for Dd2 compound **3** and **11** had the lowest and highest values of 15 and 183 respectively (Table 3). The mean *SI* values for both strains ranged from 17 to 156.

**Table 3:** CC_50_s and selectivity indices of piperidine analogues on LLC-MK2 monkey kidney epithelial cells

Compound code^a^	CC_50_ (μg/mL)	SI on 3D7	SI on Dd2	Mean SI
1	187.5	22.17	32.91	27.54 ± 7.59
2	187.5	43.51	31.37	37.44 ± 8.58
3	62.5	19.45	15.16	17.31 ± 3.0
7	375	93.67	148.34	121.01 ± 38.7
8	93.75	21.14	71.67	46.41 ± 35.7
9	250	149.52	162.97	156.25 ± 9.5
11	187.5	74.49	182.03	128.26 ± 76.1
12	187.5	55.52	67.39	61.46 ± 8.39
13	375	63.71	69.70	66.71 ± 4.24

Chemical classes of compounds:- ketones: **1**–3 and alcohols: **7**–**12**. ^a^ Compounds **4**, **5**, **6** and **10** not tested for cytotoxicity due to low activity in either the primary screen (< 50% inhibition) and or the secondary screen (IC_50_ > 10 μg/mL). Classification of cytotoxicity: CC_50_ < 1 μg/mL (high cytotoxicity), CC_50_ 1 μg/mL-10 μg/mL (moderate cytotoxicity), CC_50_ 10 μg/mL-30 μg/mL (mild cytotoxicity) and CC_50_ > 30 μg/mL (no observable cytotoxicity) [25]. Selectivity Index (SI) = CC_50_ on LLC-MK2 cells/IC_50_ on *P. falciparum*. Mean SI: geometric mean of the Selectivity Indices of the 3D7 and Dd2 parasite strains. Cut-off point for selectivity: SI > 10 (non-toxic); SI ≤ 10 (toxic) [20]

## Discussion

No new chemical class of antimalarials has been introduced to clinical practice since 2006 [20] whereas presently there is a threat of emergence of resistance to artemisinins with delayed parasite clearance by some ACTs, the last efficacious therapeutics approved for use against malaria [3]. Hence the need to discover and develop new efficacious molecules remains an active area of research. In this study, three 1, 4-disubstituted piperidines (**7**, **8**, **11**) all alcohols, were identified which were more active on the resistant Dd2 strain with lower IC_50_s than on the 3D7 strain; these analogues also showed very high selectivity for the parasite with *SI* values ranging from about 21 to 182. These findings demonstrate the potential of these piperidines as candidate antimalarial leads.

In the primary screen at a fixed concentration of 40 μg/mL, nine (60%) of the 14 piperidines produced over 80% inhibition (Table 1). This high proportion of compounds with fairly high activity is a good preliminary indication of the antiplasmodial bioactivity in the piperidine parent structure. Screening at a single concentration likely to show activity is a useful strategy which identifies active compounds early in the drug discovery pipeline, albeit in vitro in this case. This eliminates wasteful extensive screening of compounds of low interest. In the secondary screen, eight of the nine compounds showed high antiplasmodial activity. Seven of these eight compounds also showed high activity in both the two screens showing consistency in the results of the two experiments, further confirming their potential as candidate antimalarial leads. The promising analogues (**7**, **8**, **11**) identified in this work qualify for further investigation in vivo in an animal model of malaria. These compounds may be classified as antiplasmodial hits based on their high selectivity for the parasite over the mammalian cell. Their CC_50_ and *SI* values are far above the cut –off values for cytotoxicity of 30 μg/mL [19] and *SI* > 10 respectively [20] as shown on Table 3. These high *SI* values indicate very low risk of toxicity to mammalian cells. This high safety margin is not unexpected as several piperidine derivatives are being used clinically for various diseases [9], although toxicity depends on molecular structure.

Considering the activity of the compounds with respect to the present data, one can find active compounds in each of the three chemical classes i.e. ketones (**2**, **3**), alcohols (**7**, **8**, **9**, **11**, **12**) and amine (**14**). Overall alcohols exhibited the highest activity (among which are the hits) in the primary and secondary screens, the single most active and most selective compound on the resistant strain being a methanol analogue (**11**). The high activity recorded across the three chemical classes suggests that the activity is accounted for largely by the common core structure, the activity of which is modified by the substituent groups shown on Table 1.

**Fig. 2:**
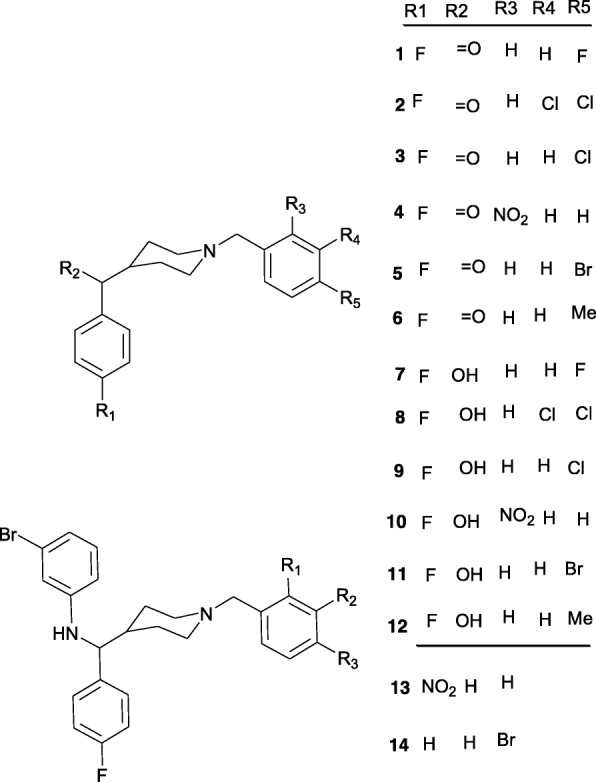
Structures of 1, 4-disubstituted piperidines

The high activity of the alcohols suggests the hydroxyl group at C-7′ in the methanol analogues (Fig. 2) is contributing greatly to the activity of these analogues. Also, the methanol analogues were the least cytotoxic as seen from their relatively higher *SI* values (Table 3). These compounds were designed as 1, 4-disubstituted piperidine analogues inspired by spipethiane, a molecule with a restricted conformation due to spirofusion in its structure and is selective for sigma-1 receptors which elicit diverse biological effects [12]. Given the presence of a piperidyl fragment in spipethiane, the latter may well turn out to be an antiplasmodial agent. The high activity displayed by these compounds supports previous reports in literature. A chemical series of 44 synthetic 1-phenethyl-4-aminopiperidine derivatives screened against *P. falciparum* K1 resistant strain demonstrated very high antiplasmodial activity; 33 of these compounds had IC_50_ values less than 5 μg/mL with 13 of them between 0.17 μg/mL and 0.91 μg/mL. These compounds were equally fairly selective for the parasites [21]. Several substituted piperidines display a wide range of biological activities like antiviral, antidepressants, cytotoxicity and antimalarial [22]. Also high antiplasmodial activity has been observed in plant derived piperidines e.g. febrifugine isolated from the roots of *Dichroa febrifuga*. Synthetic analogues of febrifugine devoid of the piperidine ring showed decreased antiplasmodial activity suggesting that the activity of febrifugine and its analogues is partly due to the piperidine ring [23]. The same finding was recorded following conversion of some natural piperidines isolated from *Senna spectabilis* to semisynthetic derivatives lacking the piperidine core [24], further illustrating the significant contribution of this core to bioactivity of molecules which posses it.

## Conclusions

Based on the antiplasmodial activity and selectivity indices, three antiplasmodial hits have been identified from the 14 synthetic piperidines screened. The activity of the 1, 4-disubstituted piperidine structure is modulated by the substituent groups particularly the hydroxyl group at C-7′ in the methanol analogues. These findings demonstrate the potential of these synthetic piperidine analogues as candidate antimalarial leads, justifying further exploration using chemistry approaches and biological screening with in vivo studies in an animal model of malaria which may yield efficacious and safe antimalarial leads.

## Abbreviations

ACT: Artemisinin based combination therapy
CC_50_: Cytotoxic concentration for 50% of population of cells
IC_50_: Inhibitory concentration for 50% of population of cells
MTT: Tetrazolium
NBT: Nitroblue tetrazolium
PES: Phenazine ethosulfate
pLDH: Parasite lactate dehydrogenase
SI: Selectivity index
